# Natural Products as a Source for New Leads in Gout Treatment

**DOI:** 10.1155/2020/8274975

**Published:** 2020-05-15

**Authors:** Samuel M. Silvestre, Paulo J. S. Almeida, Reda El-Shishtawy

**Affiliations:** ^1^CICS-UBI—Health Sciences Research Centre, University of Beira Interior, Covilhã, Portugal; ^2^CNC—Center for Neuroscience and Cell Biology, University of Coimbra, Coimbra, Portugal; ^3^Chemistry Department, Faculty of Science, King Abdulaziz University, Jeddah 21589, Saudi Arabia

Gout is a common inflammatory arthritis characterized by elevated plasmatic levels of uric acid. Several risk factors were identified, including diets rich in seafood and meat, alcohol consumption, and obesity. In this disease, when uric acid levels in the body increase due to increased formation and/or decreased excretion, the solubility limits of sodium urate are exceeded and precipitation occurs, particularly within joints, synovial fluid, and periarticular tissues. The deposits of urate crystals can initiate attacks of acute painful gouty arthritis which can evolve to chronic gout when permanent erosive joint deformity emerges. The estimated prevalence of this condition is increasing in many developed countries, particularly in men and postmenopausal women. Therefore, preventive and therapeutic strategies have been considered to avoid and treat this disease. In this context, the control of gout has been mainly performed by reducing the inflammation (e.g., with colchicine and nonsteroidal anti-inflammatory drugs), as well as by reducing the uric acid formation (e.g., using allopurinol and febuxostat) and by favouring its excretion (e.g., with probenecid and sulfinpyrazone). However, these approaches only have relative success and can originate several side effects, particularly in chronic use. For these reasons, over the years, researchers have been searching for alternative therapeutic strategies, particularly involving the use of natural products. Therefore, this Special Issue aimed to collect original research as well as review articles and meta-analysis addressing the use of natural products as well as extracts or isolated compounds in gout control. Accordingly, researchers were invited to contribute with manuscripts with *in vitro*, *in vivo*, and clinical studies focusing not only on the effects of natural products on the control of uric acid levels but also on potential treatments to reduce the gouty inflammatory arthritis. The topics of this Special Issue included: (1) sources of natural products used in the treatment of gout; (2) isolation and characterization of natural products useful in gout control; (3) natural products which increase uric acid excretion and/or reduce uric acid absorption; (4) natural products with antioxidant and anti-inflammatory effects in gout; (5) natural products which reduce uric acid biosynthesis or enhance its degradation; (6) comparison of natural and synthetic drugs in gout treatment; (7) safety of gout control with natural products; (8) chemical modifications of natural products useful in gout control to improve their efficacy and/or safety. For this Special Issue, eleven articles were published which are briefly described as follows.

The majority of the published works of this Special Issue focuses on plant extracts from different regions of the world, and their potential interest in gout control was explored by *in vitro* and/or *in vivo* studies, including xanthine oxidase inhibition and/or *in vivo* antihyperuricemic effect. In some cases, a partial phytochemical characterization was performed and potential mechanisms of action were explored. Of these, several works involve natural products from traditional Chinese medicine.

Abu Bakar et al. reported the optimization of conditions to extract phytochemical compounds from *Euphorbia hirta* L. (Ara Tanah) aiming to improve the antigout activity of extracts from the whole plant excluding roots. For this, response surface methodology and liquid-chromatography mass spectrometry analysis were performed. The authors established the best extraction conditions for total flavonoid content, total phenolic content, and *in vitro* xanthine oxidase inhibitory activity as well as the optimized levels of these compounds and its bioactivity. In addition, the main phytochemical compounds in the optimized *E. hirta* extract were determined, also aiming to be useful for further development of antigout products.

The phytochemistry, antioxidant activity, antiproliferative effect, and acute toxicity of a methanolic extract of the roots of two Moroccan Aristolochia species (*Aristolochia baetica* and *Aristolochia paucinervis*) were explored by Bourhia et al. These plants have been largely used in folk medicine to treat several diseases, including rheumatologic conditions, namely, those caused by hyperuricemia. These authors found that the two studied plants contain different classes of secondary metabolites, including polyphenols and flavonoids. In addition, radical scavenging effects were observed as well as cytotoxicity in cancer cell lines. In an *in vivo* study, no signs of toxicities nor mortalities were observed on oral-treated mice with 2000 mg/kg of the two investigated exacts.

Tseuguem et al. demonstrated that aqueous and methanol leaf extracts of *Paullinia pinnata* (Sapindaceae), a plant used to treat various diseases including arthritis, improve monosodium urate-induced gouty arthritis in rats. In addition, they evidenced the analgesic, anti-inflammatory, and antioxidant effects of these extracts by *in vivo* studies. Specifically, these authors demonstrated that both extracts significantly reduced monosodium urate-induced inflammation and hyperalgesia in both ankle and paw. In addition, a significant decrease of synovial myeloperoxidase was observed as well as nitric oxide and malondialdehyde reductions in serum, spinal, and left and right hemispheres and an increase of superoxide dismutase activity.

The beneficial effects of a macroporous resin extract of *Dendrobium candidum* leaves in rats with hyperuricemia induced by a high-purine diet were evidenced by Lou et al. In fact, after administration of this extract during 9 weeks to model rats, liver and kidney function biochemical parameters, especially serum uric acid levels, were significantly improved. In addition, a reduction of xanthine oxidase and adenosine deaminase activities in liver was observed. Moreover, according to histological analysis, this extract significantly prevented kidney and liver from damage and intestinal injury. Furthermore, a reduction in inflammation in these hyperuricemic rats by inhibiting the expression of both NF-*κ*B and TLR4 proteins was also reported.

Tu-Teng-Cao is a drug used in traditional Chinese medicine which has been widely applied in the clinical treatment of arthritis. In this context, Yao et al. evidenced that a Tu-Teng-Cao extract alleviates monosodium urate-induced acute gouty arthritis in rats by inhibiting uric acid and inflammation. Indeed, the treatment significantly attenuated the degree of ankle swelling, inflammation, and dysfunction index as well as the levels of proinflammatory cytokines in the joint fluid of the rat model of acute gouty arthritis. In addition, significant antihyperuricemia activity was also evidenced, and histological evaluation showed that the Tu-Teng-Cao extract relieved several signals of pathological damage. Moreover, this extract alleviated swelling, inflammation, and bleeding of the renal corpuscle and convoluted tubules of rats.

Huzhentongfeng is an extract from four Chinese medical herbs used in the treatment of gout. The suppressive effect of this extract on experimental gouty arthritis was explored by Wu et al. by means of *in vivo* and *in vitro* experiments. The investigators of this study observed that Huzhentongfeng could significantly suppress the paw swelling and neutrophil infiltration induced by monosodium urate intra-articular injection in rats compared with the control group. In addition, inhibition effects on the secretion of several inflammatory cytokines (IL-1*β*, IL-6, and TNF-*α*) were evidenced. Moreover, this product could prevent the oligomerization of ASC adapter proteins and had antioxidant effects in cell-free and cell-based tests.

Qu-Zhuo-Tong-Bi is an empirical traditional Chinese medicine prescription for the clinically treatment of acute gouty arthritis without serious adverse effects. In this context, Lv et al. studied the anti-inflammatory and analgesic effects of this product on acute gouty arthritis and the recurrent attack in model rats, as well as potential underlying mechanisms of action. These researchers observed that Qu-Zhuo-Tong-Bi extracts can suppress ankle swelling and synovial inflammation in the monosodium urate-induced gouty arthritis rat model, alleviate the acute attack, and prevent the recurrent attack of gouty arthritis. In addition, this treatment significantly decreased both mRNA and protein levels of NLRP3, as well as the production of IL-1 and TNF-*α* in the ankle joint. Therefore, it was concluded that Qu-Zhuo-Tong-Bi may be a promising herbal formula for the prevention and treatment of gouty arthritis in humans.

The use of isolated compounds from natural and semisynthetic origin is also presented in two articles of the Special Issue.

Wortmannin is a steroidal metabolite associated with several bioactivities, including anti-inflammatory effect. However, the mechanisms of action of this compound are not completely explored. Therefore, Mehran et al. presented multispectroscopic and molecular docking studies of the binding interaction between wortmannin and calf thymus DNA. Considering the observed results, these authors suggested that this molecule may exert its biological effects, at least in part, via interaction with DNA. In addition, it was demonstrated that wortmannin interacts with this biomolecule in the non-intercalating mode, which is considered as a new mechanism of action.

A review article, from Serrano et al., presented an integrated overview of recent studies with focus on isolated molecules with *in vitro* xanthine oxidase inhibition and *in vivo* hypouricemic effect on animal models. The analysis of data collected from the past six years demonstrated that a large number of compounds are being explored in *in vitro* studies and also in *in vivo* biological evaluations, and very promising molecules are being developed. In addition, it was verified that molecules from natural sources or their mimetics and semisynthetic derivatives constitute the majority of compounds being explored at the moment by means of *in vitro* and *in vivo* studies. Furthermore, as frequently the observed *in vitro* XO inhibition results do not have a clear correspondence with the *in vivo* hypouricemic effects, it is necessary to perform other complementary studies to better establish the pharmacological profile of these molecules under development.

Finally, the analysis of existing clinical data on the use of natural products in gout control was also explored in two review articles.

The effectiveness of cherries in reducing uric acid and gout was studied by Chen et al. in a systematic review of the clinical evidence in this point. After application of the search strategy, six clinical studies reporting decreases in the incidence and severity of gout following the ingestion of cherries or products containing cherries were included in this review. Despite the described positive effects of cherry intake in gout, it was not possible to conduct a meta-analysis due to the lack of relevant studies and to the high degree of variation in the methodologies and metrics used in the studies included in this review. Therefore, additional more rigorous and larger clinical trials are needed, including long-term follow-up studies.

A systematic review and meta-analysis of randomized controlled trials on natural product dietary supplements on patients with gout was presented by Yang et al. After application of the search strategy and establishment of the clinical outcomes, nine randomized controlled trials were included in the review, and meta-analysis concerning the efficacy and safety of these supplements were performed. The authors observed that natural product dietary supplements appeared to be superior to control groups in affected joint pain, swelling, and activity limitation, while not in decreasing seric uric acid and C-reactive protein levels or the incidence of adverse effects. However, due to poor trial quality and the absence of standardized evaluation criteria, the current existing evidence is insufficient to allow a definitive statement about the efficacy and safety of these supplements. Hence, further larger and more rigorously designed randomized controlled trials are clearly needed in the future.

Thus, with contributions from research groups from diverse countries, this Special Issue presented recent experimental findings and reviews on natural and semisynthetic products with relevant potential for the prevention and treatment of gout.

